# Transcranial electric stimulation seen from within the brain

**DOI:** 10.7554/eLife.25812

**Published:** 2017-03-28

**Authors:** Angel V Peterchev

**Affiliations:** 1 1 Department of Psychiatry and Behavioral Sciences, Duke University, Durham, United Statesangel.peterchev@duke.edu; 2 Department of Biomedical Engineering, Duke University, Durham, United States; 3 Department of Electrical and Computer Engineering, Duke University, Durham, United States; 4 Department of Neurosurgery, Duke University, Durham, United States

**Keywords:** transcranial electric stimulation, computational current-flow model, intracranial recordings, Human

## Abstract

Computer models can make transcranial electric stimulation a better tool for research and therapy.

**Related research article** Huang Y, Liu AA, Lafon B, Friedman D, Dayan M, Wang X, Bikson M, Doyle WK, Devinsky O, Parra LC. 2017. Measurements and models of electric fields in the *in vivo* human brain during transcranial electric stimulation. *eLife* **6**:e18834. doi: 10.7554/eLife.18834

The human brain seems well protected, encased within the skull. Yet something as simple as placing a pair of wet sponges onto someone's head and sending a weak electric current between them can actually alter the brain's activity. A refined version of this method – known as transcranial electric stimulation – has attracted considerable interest and is now being used to probe the workings of the brain and develop treatments for medical conditions such as depression, epilepsy or stroke.

Transcranial electric stimulation (or TES for short) has parallels with conventional drug treatments in the sense that delivering an electric field to the brain is analogous to delivering drug molecules into the body. So, just as it is important to know how the human body affects an administered drug (a field of research that is known as pharmacokinetics), in TES we need to know how much of the current applied to the scalp actually enters the brain, and where this current goes.

The 'pharmacokinetics of TES' remains contentious (Underwood, 2016), but is important for several reasons. First, it allows us to relate findings from experiments in which brain tissue from animals is stimulated directly to findings obtained via noninvasive applications in people. Second, it helps researchers optimize the process in order to target specific regions of the brain. Third, it enables researchers to compensate for the differences between individuals, and to standardize the exposure that they receive.

The only established approach for estimating the dose of TES delivered to an individual relies on a three-dimensional model of the subject's head that includes its different tissues and the attached electrodes, which is fed into a computer simulation (Figure 1). Such models have been available for some time (Datta et al., 2009), but they had been validated only partially and indirectly in humans or other primates (Edwards et al., 2013; Lee et al., 2015). Moreover, there are uncertainties about the electric properties of the tissues in these models.

**Figure 1.**
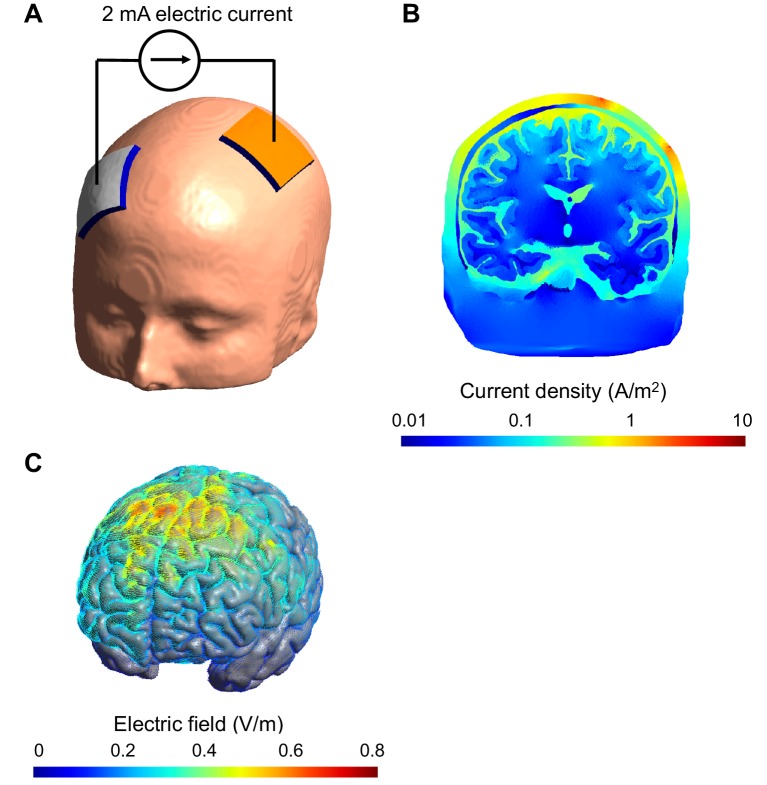
Computational model of the electric field and current produced in an individual's head during transcranial electric stimulation. (**A**) Electrodes (white and orange rectangles) are attached to the scalp and electric current is applied; the model of the head shown here is derived from a structural MRI scan. (**B**) Simulation showing the electric current per unit area (current density) in a section of the brain during transcranial stimulation: this image shows the scalp (outermost layer), skull, cerebrospinal fluid, gray matter and white matter. The highest current density values in the brain (blue) are 100-fold lower than those in the scalp (red). The high resistance of the skull means that the majority of the current is shunted in the scalp. The cerebrospinal fluid is highly conductive and this takes current away from the brain too. (**C**) Simulation showing the electric field on the surface of the brain. For this configuration, the electric field is strongest between the two electrodes. The model was created and visualized with the free SimNIBS software package (http://simnibs.de; Windhoff et al., 2013).

Now, in eLife, Lucas Parra and colleagues – including Yu Huang and Anli Liu as joint first authors – report how they have addressed these issues by combining elaborate computational modeling with recordings taken within the brains of ten people undergoing surgery for epilepsy (Huang et al., 2017). This sample size markedly exceeds that of other similar measurements (Opitz et al., 2016), and the three-dimensional models used are highly sophisticated too. Leveraging this setup, Huang et al. provide the most extensive and direct estimates of the TES electric field to date. They also confirm that computational models of TES can accurately recreate the electric field generated in a real brain.

Huang et al. – who are based at City College of the City University of New York, New York University School of Medicine and the Mayo Clinic – provide practical insights that should help others to implement the models as well. For accurate results, the individual scan should capture the entire head, from neck to crown. This is not the convention in clinical imaging, which currently only focuses on the brain, but Huang et al. get round this limitation by splicing the bottom portion of a standard model of a head onto the individual scans. To do this, the images must be properly cropped and morphed, though this feature has yet to be added to publicly available electric field modeling software.

Including a compartment for the cerebrospinal fluid (the colorless liquid that surrounds the brain) also makes the models more accurate. Appropriate imaging and image analysis methods are required to capture this layer as well as the skull, which are both quite thin (see Figure 1B). However, modelers can breathe a sigh of relief, because the data suggest that the different layers within the skull can be omitted from the models without significantly impacting their accuracy. The way that conductivity changes depending on the orientation of the current in the brain's white matter can similarly be ignored, at least for the mostly outer regions of the brain explored so far by Huang et al.

This work also underscores the present limitations of modeling. It is still uncertain exactly what values for tissue conductivity should be used, and whether it is acceptable to use the same values for everyone. Addressing this question requires further studies likely involving a range of techniques. For example, there are promising efforts to measure tissue conductivities directly during surgery (Koessler et al., 2017), or with other noninvasive techniques (Chauhan et al., 2017).

Even without making the absolute electric field estimates more accurate, existing modeling approaches and software appear suitable for measuring the relative strength of stimulation across brain regions, and predicting how an individual's anatomy might affect this. Indeed, the National Institutes of Health now requires that researchers applying for certain grants "use realistic head modeling" to characterize what electric field is delivered across the brain (NIH, 2017). All in all, it seems that the time is now right for wider adoption of 'pharmacokinetics' of transcranial brain stimulation.
